# Investigation of halloysite nanotubes and Schiff base combination with deposited copper iodide nanoparticles as a novel heterogeneous catalytic system

**DOI:** 10.1038/s41598-021-02991-9

**Published:** 2021-12-08

**Authors:** Mansoureh Daraie, Donya Bagheri, Masoume Malmir, Majid M. Heravi

**Affiliations:** Department of Chemistry, School of Physics and Chemistry, Alzahra University, Vanak, PO Box 1993891176, Tehran, Iran

**Keywords:** Catalysis, Green chemistry

## Abstract

The design, preparation and characterization of a novel composite based on functionalization of halloysite nanoclay with Schiff base followed by immobilization of copper iodide as nanoparticles is revealed. This novel nano composite was fully characterized by utilization of FTIR, SEM/EDX, TGA, XRD and BET techniques. This Cu(I) NPs immobilized onto halloysite was successfully examined as a heterogeneous, thus easily recoverable and reusable catalyst in one of classist organic name reaction so-called “Click Reaction”. That comprised a three component reaction of phenylacetylene, α-haloketone or alkyl halide and sodium azide in aqueous media to furnish 1,2,3‐triazoles in short reaction time and high yields. Remarkably, the examination of the reusability of the catalyst confirmed that the catalyst could be reused at least six reaction runs without appreciable loss of its catalytic activity.

## Introduction

Clay minerals are phyllosilicate, which believed, have been existed since the life began. They have particular morphologies, thus, enabling to interact with various molecules to generate nanocomposites with high molecular diversity^1,2^. Among them, halloysite nanotubes (HNTs) are known as aluminosilicates, of the kaolin group. They mainly have hollow tubular morphology with chemical formula Al_2_Si_2_O_5_(OH)_4_·nH_2_O. HNTs which are a dioctahedral 1:1 clay mineral found some in soils, particularly those collected from wet tropical and subtropical areas. These regions and weathered igneous and non-igneous rocks, are place chiefly in New Zealand, Mexico, Brazil USA, Australia and China. Depends on their changeable deposit, HNTs are existed in different hydration state, characteristic sizes, and purity status. Interesting to know that halloysite initially was discovered by the Belgian geologist Omalius d’Halloy in 1826. Among the mineralogist he is also known as M. Berthier. Nowadays, halloysite has received much attention of synthetic chemists and stirred up the interest the scientific community^3–5^. Due to unique and elegant chemical and interesting physical properties Hal, has been extensively employed in both academic and industrial research, projects, for example leading to its practical use as nanocarrier, adsorbent, and catalyst^6–8^.

From the structural points of view, naturally occurring HNTs comprises nanotubes similar to cylinders, self-installed of octahedral Al–OH sheet unites, on the inner surface, SiO_2_ bonds on the outer surface, in which the cylinders are separated from each other by a layer of adsorbed water^9–11^. Owing to this motivating structural features, and due to the presence of free hydroxyl groups on both its inner and outer surfaces, HNTs have been chemically modified. These modifications mostly have been devoted and focused on designing and preparation of effective and stable catalysts and suitable supports for being used as superior heterogeneous catalysts in different chemical transformations^12,13^.

The Schiff bases are commonly prepared by condensation of different compounds bearing carbonyl moieties with various amines. These bases can be reacted with various metals and oxo-metal cations to provide the corresponding stable metal chelates^14^. As a matter of fact these kind of chelates have been found the most applicable ones in coordination chemistry, which can be employed in the synthesis of the various agents as polymer stabilizer, and also dyes and pigments, as well as catalytic systems^15,16^. Some Schiff bases prepared from carbonyl involving aromatic compounds due to their unique electronic and steric structures are widely used as biological and metalloprotein models and asymmetric catalysts^17–19^. Schiff bases are chelated with the most of metal cations, providing the corresponding complexes. Among these metals, copper as Cu-cations have been found the best since their reaction with different Schiff bases give complexes which has been proven to be most efficiently and practically applicable catalysts. Several complexes of Schiff bases as catalytic agents, have been found readily accessible, highly effective and more important very stable in a wide range of organic transformations. Due to their great stability, they are exceptionally useful the catalysts of choice, for the organic reactions, conducted at high temperature (> 100 °C), even under wet conditions^20–24^.

Click chemistry as one of the most prevalent reactions, originally introduced by Sharpless and coworkers in 2001, is performed with high selectivity under mild conditions. This reaction has been used in a wide range of research areas such as, polymers, drug discovery and supramolecular chemistry^25–27^. Among click reactions, Huisgen 1,3-dipolar cycloaddition is the most well-known example, in which organic azides and terminal alkynes are combined to provide 1,4-disubstituted 1,2,3-triazole derivatives. 1,2,3-triazoles compound is an significant heterocyclic category of organic compounds with extensive range of applications^28,29^. More recently, novel methods have been used to promote click reaction, including the incorporation of Cu metal into some organic and inorganic materials for preparing heterogeneous or homogeneous catalytic systems^30–37^. Despite all the achievements, some limitations such as high reaction temperature and duration, use of toxic solvents and high amount of the catalyst have been still unsolved. It is therefore necessary to promote efficient methods.

In continuation of interest on using of functionalized HNTs for immobilizing catalytic active species^38–40^ and our continuous interest in click reaction from different points of view^41–45^, herein, we wish to report the preparation of a novel Schiff base-halloysite hybrid system, as a catalytic support for immobilization of CuI nanoparticles and reveal our investigation on the aforementioned, hybrid catalyst, CuI@HNT-TSC-PC in one of the classist organic transformations so called “Click Reaction”.

## Experimental

### Materials and instruments

All materials and solvents, such as, Halloysite (Kaolin) CAS Number: 1332-58-7; (3-chloropropyl)trimethoxysilane 97%; CAS Number: 2530-87-2; Thiosemicarbazide 98%; CAS Number 79-19-6; Pyridine-2-carbaldehyde 99%; CAS Number: 1121-60-4; Triethylamine (TEA) 99%; CAS Number: 121-44-8; CuI 99.5%; CAS Number: 7681-65-4; were purchased from Sigma-Aldrich. Toluene 99%; CAS Number 108-88-3; and Ethanol 96%; CAS Number 64-17-5; were purchased from Merck Millipore and used as received, without any further purification. The click reaction was performed by using terminal alkynes including, Phenyl acetylene 98%; CAS Number: 536-74-3; 4-ethynyltoluene 97%; CAS Number: 766-97-2; Propargyl alcohol 99%; CAS Number: 107-19-7; 2-methyl-3-butyn-2-ol 98%; CAS Number: 115-19-5, and α-haloketones; Benzyl bromide, 98%; CAS Number: 100-39-0; Benzyl chloride 99%; CAS Number: 100-44-7; Benzoyl bromide 97%; CAS Number: 618-32-6; 4-chlorobenzoyl chloride 99%; CAS Number: 122-01-0; 2-bromobenzoyl chloride 98%; CAS Number: 7154-66-7; 4-bromobenzoyl chloride 98%; CAS Number: 586-75-4; 4-methylbenzoyl bromide, 95%; CAS Number: 874-58-8; 4-methylbenzyl chloride 98%; CAS Number: 104-82-5; 4-chlorobenzyl chloride 95%; CAS Number: 104-83-6; 4-nitrobenzyl bromide 99%; CAS Number: 100-11-8; 4-methylbenzyl bromide 97%; CAS Number: 104-81-4; Iodomethane; CAS Number: 74-88-4 and Sodium azide ≥ 99%; CAS Number: 26628-22-8 purchased from Sigma-Aldrich and Merck Millipore.

The new prepared nano composite, CuI@HNT-TSC-PC was fully characterized by employing different techniques such as FTIR, SEM, EDX, XRD, TGA, and ICP-AES. Bruker Tensor 27 instrument was used for recording the FTIR spectra between 4000 and 400 cm^−1^ and the KBr pellet technique was employed: about 1 mg of the sample and 300 mg of KBr were used to prepare the pellets. SEM/EDS images were recorded by a TESCAN, VEGA 3 SEM instrument and all samples had been coated with a thin gold layer by evaporation. X-ray diffraction patterns of CuI@HNT-TSC-PC, CuI and halloysite were recorded at room temperature by using a Siemens, D5000 diffractometer with Ni-filtered Cu Kα radiation, working at 40 kV and 30 mA, at a scanning speed of 2°/min in the scan range from 5° to 80° 2*θ*. Thermogravimetric (TG) and differential thermal (DTA) analyses were conducted in a NETZSCH TG 209 F1 Iris thermo gravimetric analysis apparatus from 25 to 600 °C, under nitrogen atmosphere, at a heating rate of 15 °C/min. The BET analysis of the CuI@HNT-TSC-PC was performed using a fully automated BET surface area analyzer (Brunauer–Emmett–Teller, model: Belsorp-mini II) instrument at − 196 °C; 0.2 g of the sample was used. A Perkin-Elmer Optima 3100XL axial viewing ICP-AES equipped with a cyclonic spray chamber and a GemTip cross-flow nebulizer was used for the determination of the trace Cu elements. The CuI@HNT-TSC-PC were introduced into the ICP-AES system at a flow rate of 1.0 mL min^−1^. All products were known and identified by comparison of their physical and spectroscopic data with those of authentic compounds reported previously and found being identical and also several of products were identified by NMR analysis.

### Preparation of nano composite

#### Preparation of Cl-functionalized halloysite: HNT-Cl

To functionalize HNTs, initially, HNTs (1.5 g) was dispersed in 40 ml dry toluene and then CPTES (4 ml) was added dropwise to the mixture. The resulting suspension was refluxed at 110 °C for 24 h. At the end of the process, the resulting precipitate was separated by simple filtering, washed and dried at 80 °C overnight.

#### Synthesis of Schiff base: TSC-PC

Schiff base was prepared according to the previously reported procedure^46^. Briefly, pyridine-2-carbaldehyde (10 mmol, 1.05 ml) and thiosemicarbazide (10 mmol, 0.94 g) were dissolved in water (10 ml) and heated at 75 °C for 6 h. Upon completion of the reaction, the yellow precipitate was filtered off, and washed with EtOH (10 ml) and dried at 70 °C.

#### Synthesis of HNT-TSC-PC

Conjugation of HNT-Cl and Schiff base was accomplished through the following procedure: first, HNT-Cl (1.5 g) was well-dispersed in dry toluene by using ultrasonic irradiation for 20 min. then, Schiff base (TSC-PC) (1.5 g) and TEA (0.1 ml) were added and the resulting mixture refluxed overnight. At the end of the reaction, the obtained product was filtered off, washed with toluene and dried in oven.

#### Immobilization of CuI NPs on the HNT-TSC-PC: Synthesis of CuI@HNT-TSC-PC

**Figure 1:**
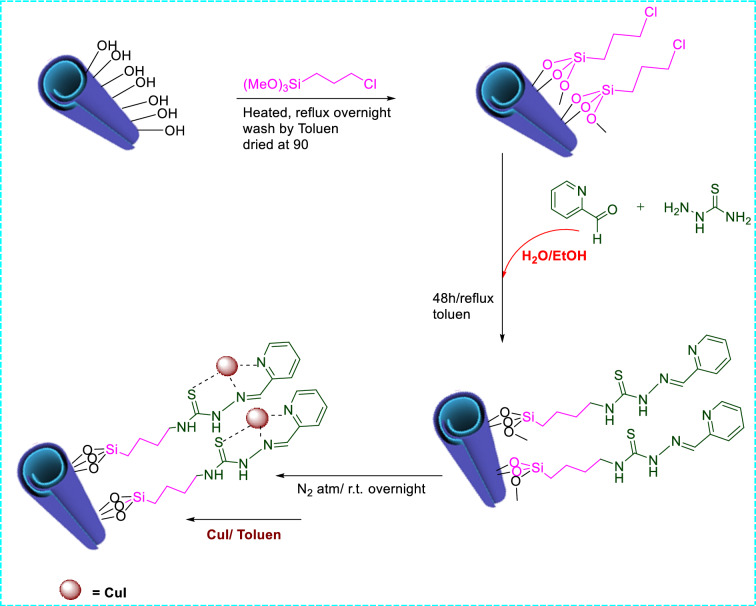
Preparation of CuI@HNT-TSC-PC.

HNT-TSC-PC (1 g) was dispersed in toluene and a solution of CuI (0.095 g, 0.5 mmol) in CH_3_CN was then added into the suspension and the obtained mixture was stirred under N_2_ atmosphere for 10 h. Finally, the product was collected, washed with toluene and dried at 70 °C for 12 h (Fig. 1).

#### Regioselective synthesis, of 1,4-disubstituted 1,2,3-triazoles via click reaction: general procedure

Alkyne (1 mmol) and α-haloketone or alkyl halide (1.0 mmol) and sodium azide (1.3 mmol) were mixed in 5.0 ml water:ethanol (1:1) mixture in the presence of CuI@HNT-TSC-PC (0.03 g) and the resulting mixture was refluxed for appropriate time. Upon completion of the reaction (monitored by TLC), the mixture was filtered off, and the filtrated was washed with deionized water and purified by recrystallization with hot ethanol. The residue catalyst was washed and dried at 80 °C for the next reaction runs.

## Result and discussion

### Catalyst characterization

**Figure 2:**
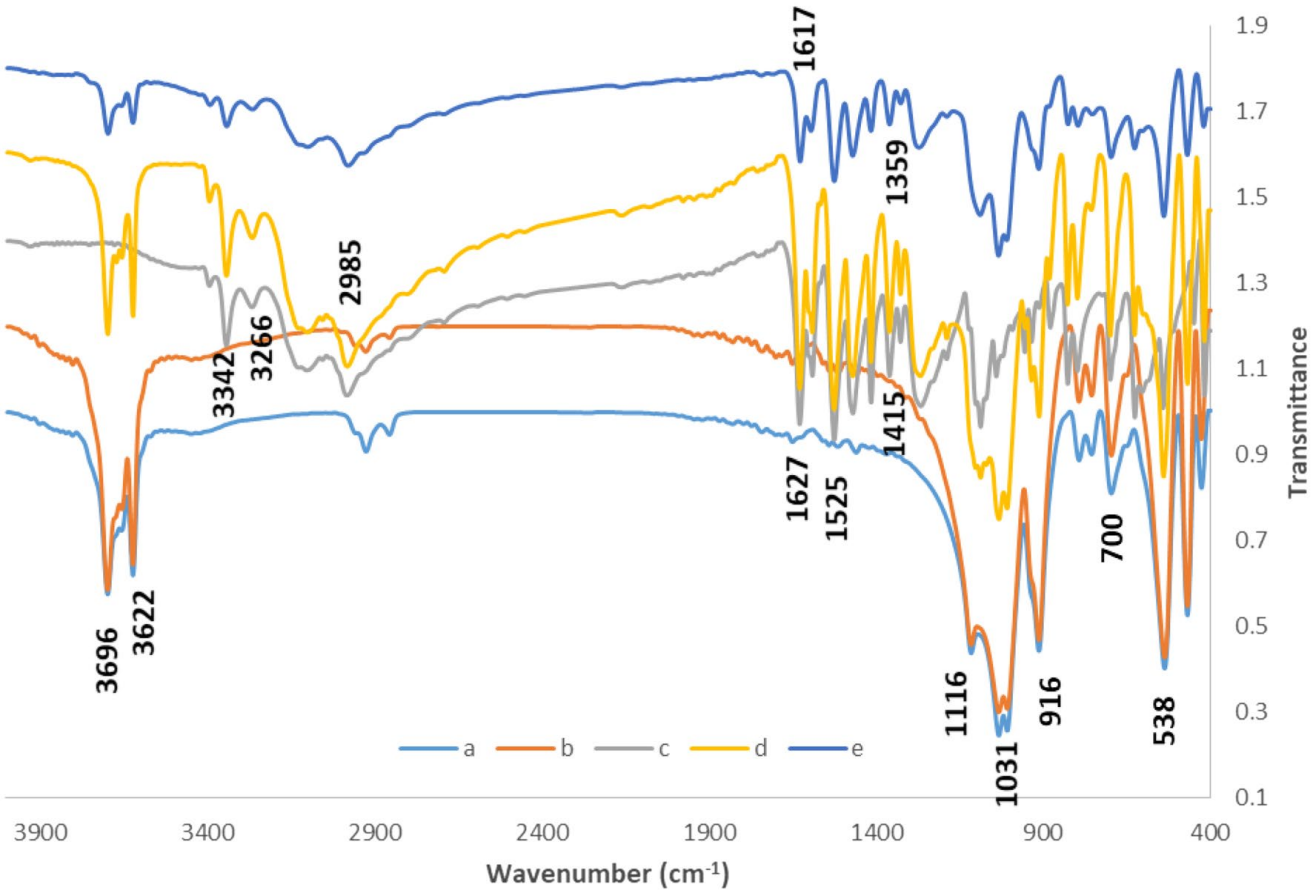
The FTIR spectra of (a) pure HNTs, (b) HNT-Cl, (c) TSC-PC, (d) HNT-TSC-PC and (e) CuI@HNT-TSC-PC.

First, to study the structure of CuI@HNT-TSC-PC and to confirm the appropriate progress of each stage of catalyst synthesis, the FTIR spectra of HNTs and the product of each step of catalyst synthesis (TSC-PC, HNT-Cl, HNT-TSC-PC, CuI@HNT-TSC-PC) were recorded (Fig. 2). The FTIR spectra of HNTs showed the characteristic bands at 1649 cm^−1^ (Si–O stretching, 539 cm^−1^ (Al–O–Si vibration), and 3621–3696 cm^−1^ (inner –OH groups)^47^. The similarity between FTIR spectrum of HNT-Cl and pure HNTs, indicates the stability of the halloysite structure after functionalization with organosilane. The FTIR spectrum of the HNT-TSC-PC showed the characteristic bands of HNTs included, 1649, 539 and 3621–3696 cm^−1^ as well as two additional band at 1326 cm^−1^, which is representative of C=S stretching and the strong absorption peaks of the C=N band at 1627 cm^−1^ confirms the successful formation of Schiff base (Fig. 2c, TSC-PC). Moreover, in the FTIR spectrum of the catalyst not only all characteristic bands were observed, but also two bands at around 1415 and 1627 cm^−1^ of Schiff base were shifted slightly to right (1359 and 1617 cm^−1^), indicates successful interaction between Cu and C=N and C=S groups.

**Figure 3:**
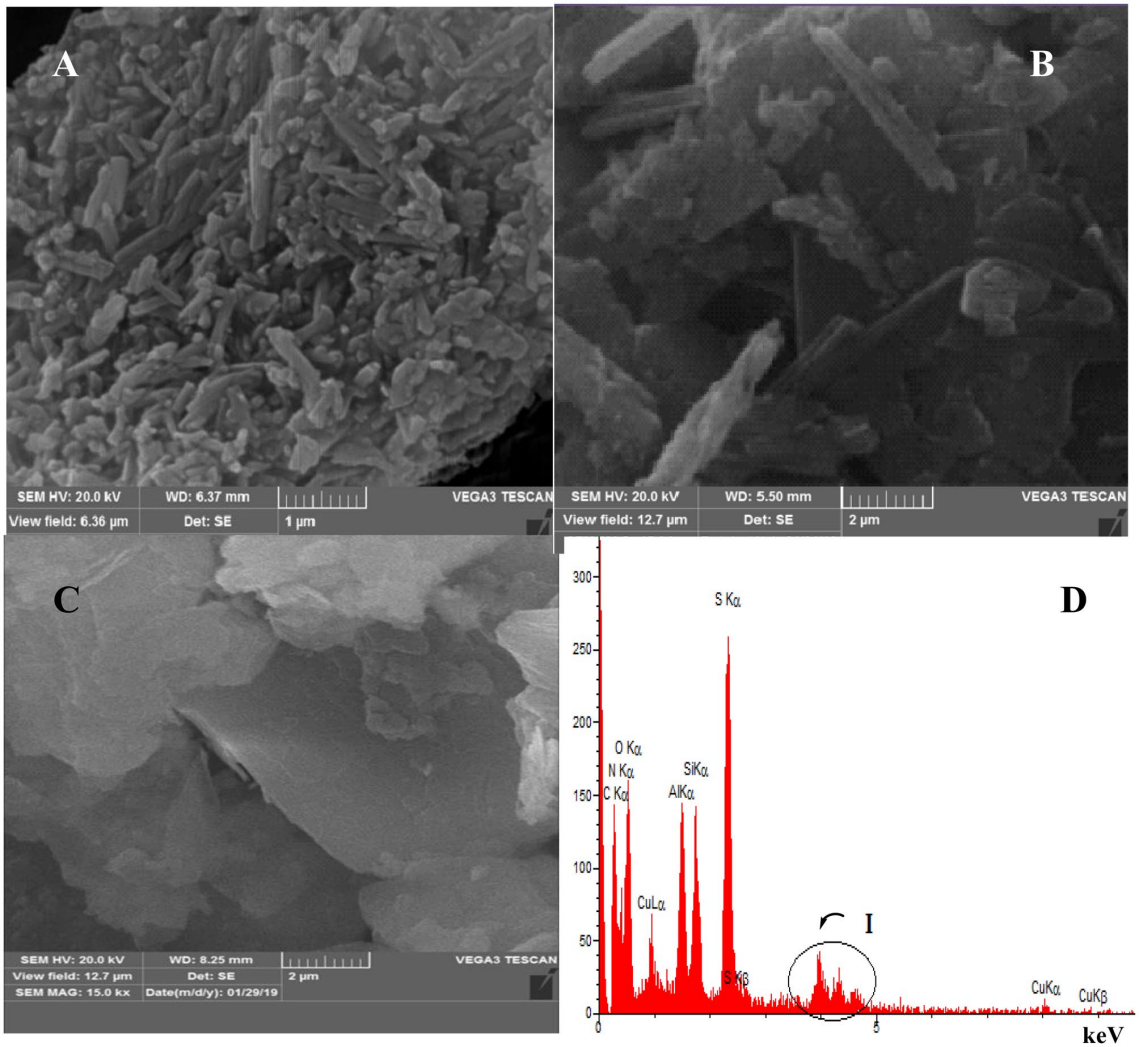
SEM images of (**A**) pure HNTs and (**B**, **C**) CuI@HNT-TSC-PC and (**D**) EDS analysis of CuI@HNT-TSC-PC.

The morphology of the catalyst was investigated by SEM/EDS techniques (Fig. 3). The SEM images of CuI@HNT-TSC-PC are shown in Fig. 3B and C. As depicted, the short tubes of HNTs closed together to form aggregates. Compared to the tubular morphology of pure HNTs (Fig. 3A), CuI@HNT-TSC-PC exhibited a distinguished morphology, which is more compact than pure HNTs. This observation can be attributed to the presence of Schiff base on the surface of HNTs and the possibility of electrostatic interactions among the tubes. This may bring the tubes closer and facilitate formation of aggregates. As depicted in the SEM image, the average diameter size of synthesized CuI@HNT-TSC-PC was 27 nm. The EDS analysis of the catalyst is presented in Fig. 3D. The observation and concentration of Si (2.81%), Al (2.98%) and O (26.83%) in the EDS of CuI@HNT-TSC-PC can indicate HNTs structure. The presence of C (27.96%), N (28.35%) and S (7.66%) atoms can confirm the incorporation of Schiff base that the high concentration observed in C and O atoms may be related to the holder. Additionally, the presence of Cu (1.48%) and I (1.93%) can be representative of CuI in the structure of the catalyst.

**Figure 4:**
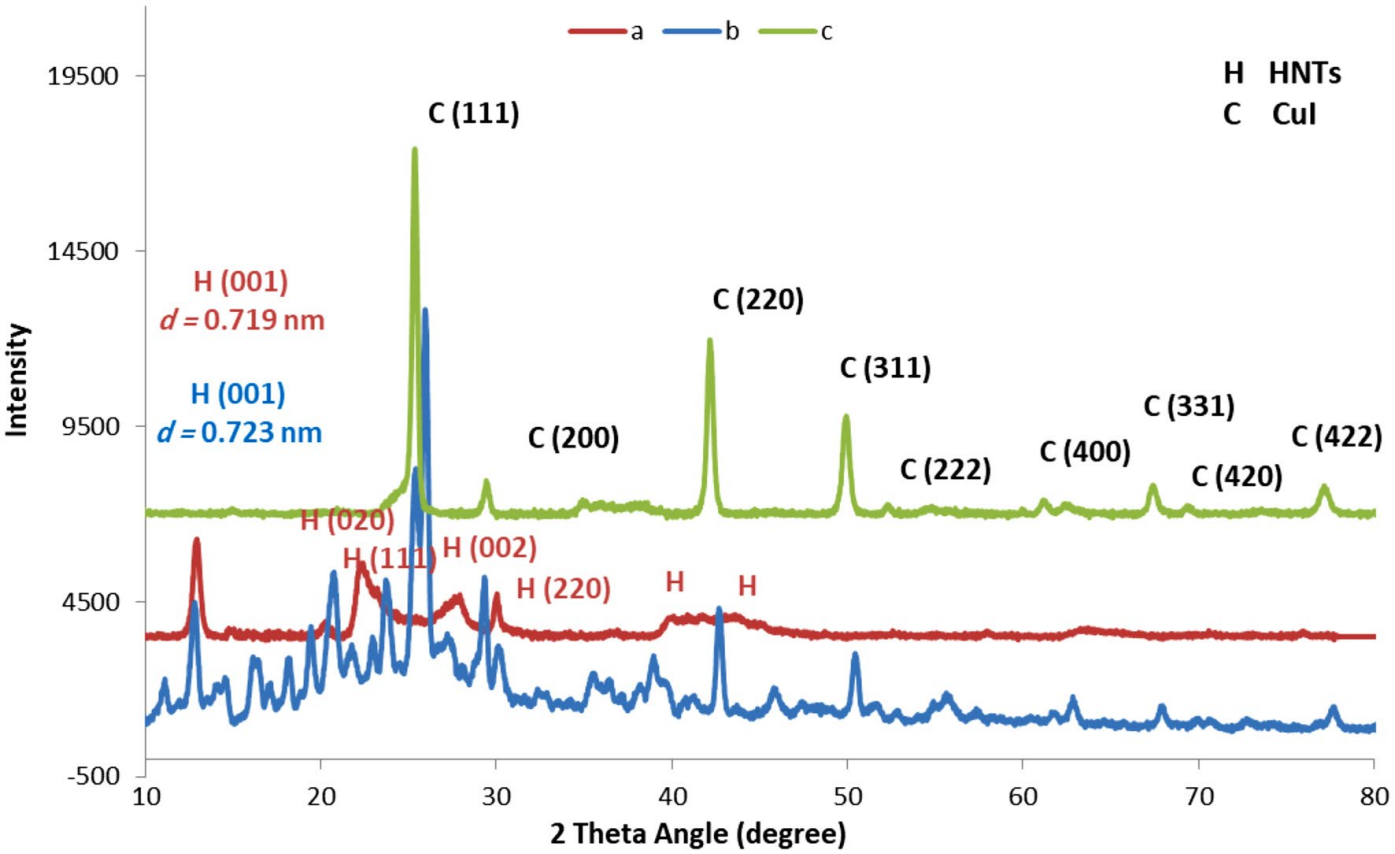
XRD patterns of (a) CuI@HNT-TSC-PC, (b) pure HNTs and (c) pure CuI.

Subsequently, the catalyst was also characterized by using XRD analysis, Fig. 4. The obtained XRD pattern was compared with that of pure CuI and HNTs, Fig. 4. Comparing all XRD patterns, it can be concluded that the XRD pattern of CuI@HNT-TSC-PC exhibits the characteristic bands of pure HNTs, the peaks observed at 2*θ* = 8°, 14°, 24°, 28°, 32°, 56° and 65° (JCPDS No. 29-1487, labeled as H)^48,49^, after CuI incorporation, the structure of HNTs did not collapse Clearly, a sharp diffraction peak is located at 24.9, corresponding to the (111) crystal planes of CuI, which shows that the CuI have good crystal structures. Comparing two XRD patterns, pure CuI and catalyst, seven peaks located at 30°, 42.9°, 46.3°, 50.9°, 52.2°, 60.2°, 67.1°, 69.0° and 76.9°, labeled as C, corresponding (200), (220), (311), (222), (400), (331), (420) and (422) crystal planes of CuI, which match well with JCPDS card (no. 01-076-0207)^30^.

Moreover, the pure HNTs show a diffraction peak at 2*θ* = 12.25° (001), which is related to its tubular morphology, high degree of disorder, small crystal size, and interstratifications of layer with various hydration states. In the XRD pattern of the catalyst, this peak is shifted to a lower 2*θ* value. The *d*-spacing of catalyst is 0.716 nm at a 2*θ* of 12.18°. The evidence of intercalation between Schiff base and the HNTs is strongly supported by the 2*θ* reductions of the increases in the basal spacing of the HNTs in catalyst, which confirms the formation of CuI@HNT-TSC-PC. In relation to the two additional diffraction peaks displayed in the XRD pattern for pure HNTs at 2*θ* of 20.06° (020) and 24.94° (002), the subsequent XRD patterns for the catalyst revealed that the pure HNTs peak at 2*θ* at around 20.06°, for the catalyst, had shifted markedly lower, and the peak at 2*θ* at around 24.94°, for the catalyst, had almost completely vanished. These results support the existence of intercalation of the organic linker into the structure of the HNTs.

**Figure 5:**
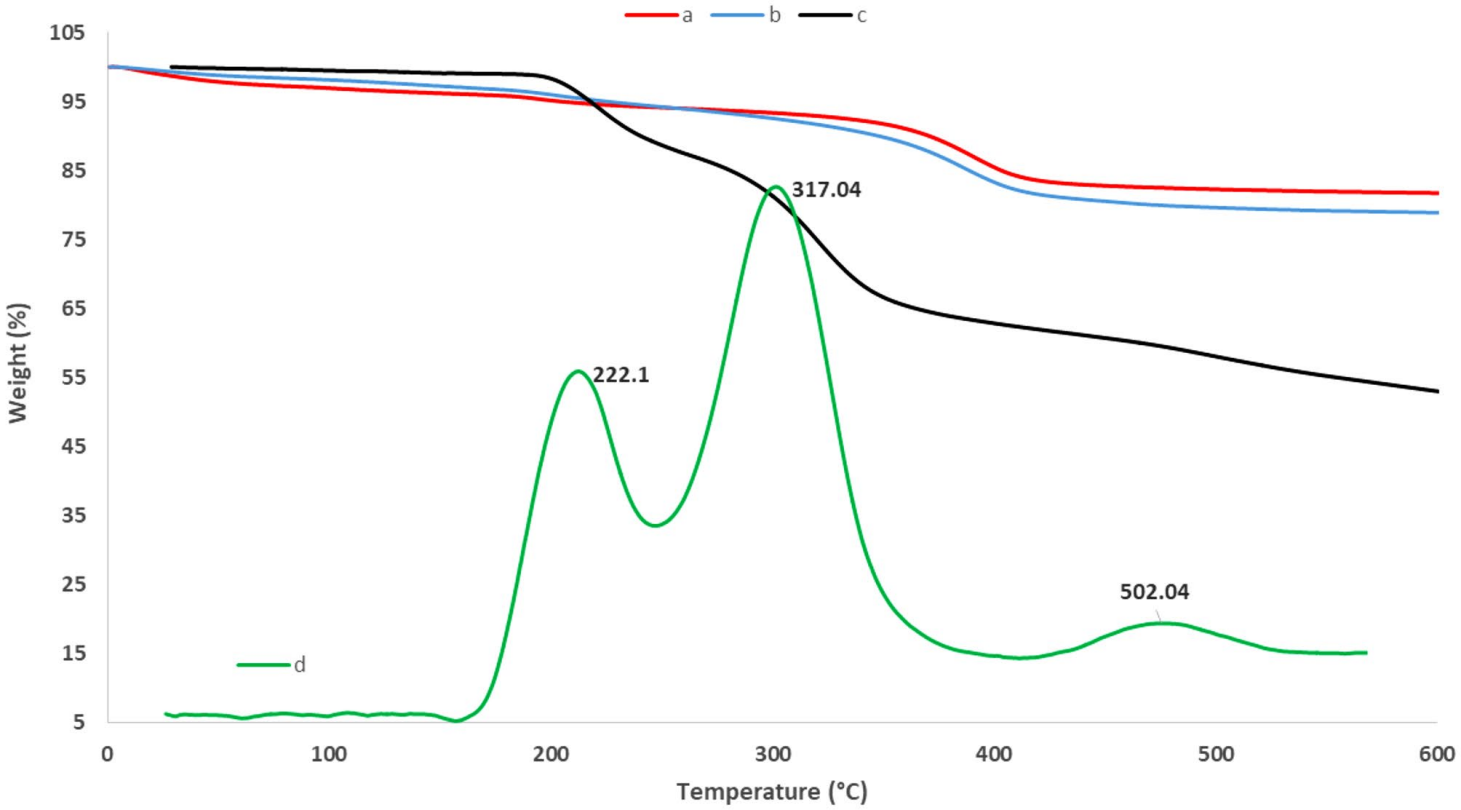
The TG analyses of (a) pure HNTs, (b) HNT-Cl, (c) CuI@HNT-TSC-PC and (d) DTGA spectra of CuI@HNT-TSC-PC.

To further characterization of the catalyst, the thermal stability of pure HNTs, HNT-Cl and CuI@HNT-TSC-PC were studied using TGA (Fig. 5). As shown, pure HNTs exhibited two weight losses, the first one, around 150 °C, is because of losing water and the second one, around 500 °C, is due to the dehydroxylation of the HNTs matrix^50^. To estimate the content of the organosilan, Cl, on the surface of HNTs, the thermogram of HNT-Cl was obtained and compared with that of pure HNTs, Fig. 5a and b. The results showed that the content of organosilan was about 4 w/w%. Next, the thermograms (TG and DTG analyses) of catalyst were recorded. According to Fig. 5c, three steps weight losses were exhibited, which are attributed to the loss of water (130 °C), decomposition of organic groups (270 °C) and dehydroxylation of Hal (480 °C). Moreover, to estimate the content of Schiff base and CuI on the catalyst, the thermogram of CuI@HNT-TSC-PC was considered and compered with that of HNT-Cl. The comparison of the thermograms of HNT-Cl and CuI@HNT-TSC-PC, showed that incorporation of Schiff base and CuI can alter the thermogram obviously, that this can be due to degradation of copper iodide. The calculation showed that the content of Schiff base was about 34.2 wt%.

**Figure 6:**
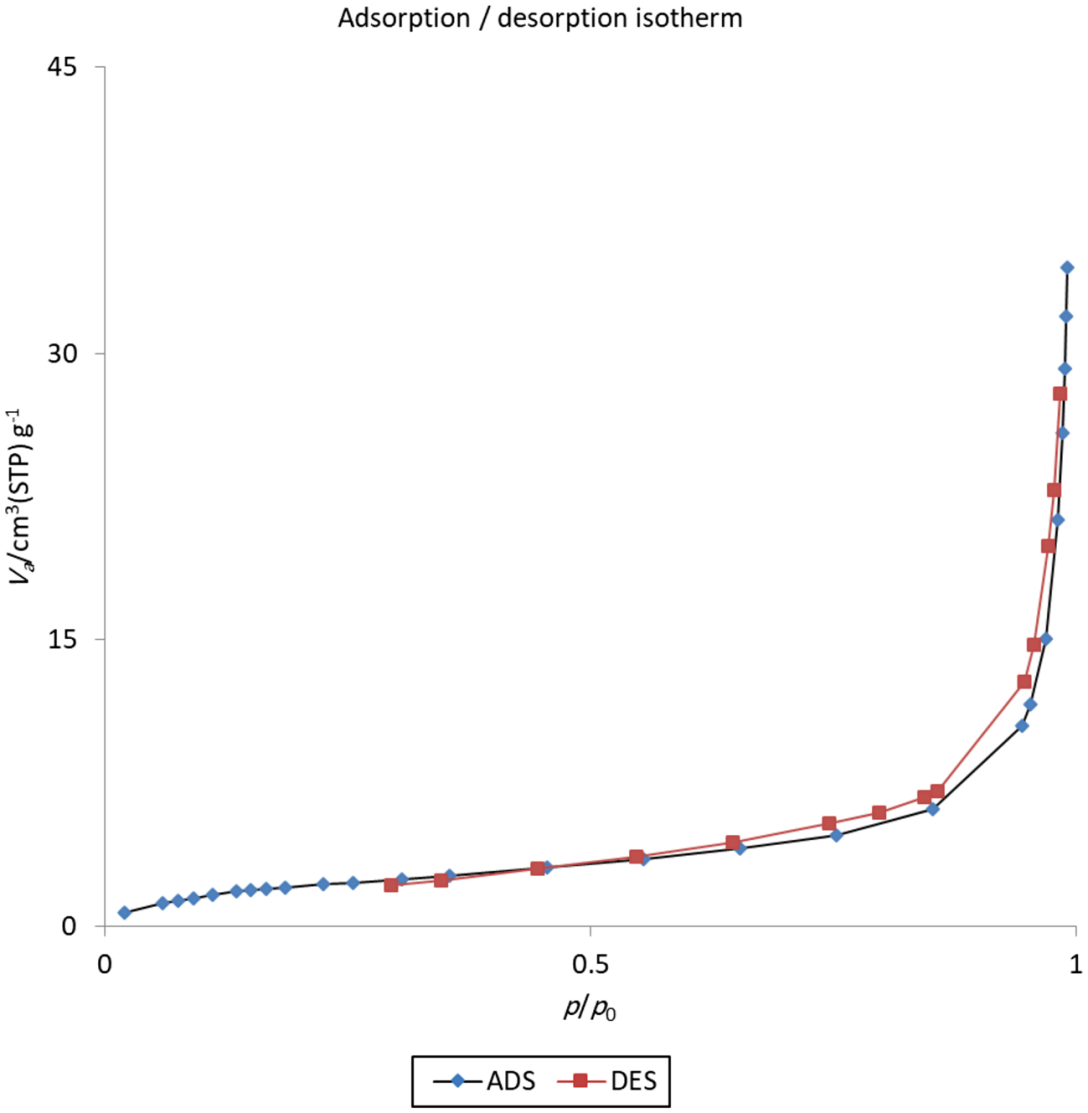
The N_2_ adsorption–desorption isotherm of CuI@HNT-TSC-PC. ADS adsorption, DES desorption.

Finally, BET analysis was performed to confirm that ligand and copper nanoparticles were located on the surface of Hal and examined the textural of the catalyst. The CuI@HNT-TSC-PC nitrogen adsorption–desorption isotherm is illustrated in Fig. 6 and Table 1. As shown, the recorded isotherm of type II isotherms is similar to pure HNTs^51^. As shown, the recorded isotherm is a type II isotherm and is similar to the pure HNTs sample. Using BET, the specific surface area of CuI@HNT-TSC-PC was measured 8.48 m^2^ g^−1^, which is much lower than the pristine Hal (51 m^2^ g^−1^). These observations indicate that ligand and CuI nanoparticles are located on the outer surface of the Hal.

**Table 1:** Textural property of CuI@HNT-TSC-PC catalyst.

*S*_BET_ (m^2^ g^−1^)	*Vm *(cm^3^(STP) g^−1^)	C	Total pore volume (cm^3^ g^−1^)	Mean pore diameter (nm)	Slope	Intercept
8.4806	1.9485	23.68	0.052189	24.615	0.5382	0.0216

Cu loading of CuI@HNT-TSC-PC was measured by ICP-AES analysis. Sample was prepared for ICP analysis as follow: CuI@HNT-TSC-PC (0.02 g) was digested in a mixture (1:3) of concentrated nitric acid and hydrochloric acid by continuous stirring for 24 h. The extract was then analyzed by ICP-AES. The Cu content of catalyst was measured at about 3.6 wt%.

### Catalytic activity

Due to the importance of 1,2,3‐triazoles for the synthesis of biologically active compounds, the catalytic activity of CuI@HNT-TSC-PC was investigated to promote click reaction. Initially, the reaction of phenylacetylene, benzyl bromide and sodium azide was selected as a model reaction and performed in the presence of various solvents as well as under solvent‐free conditions (40 mg). Pleasantly, by comparing the results obtained from running the model reaction in different solvents, it is concluded that water as a green solvent gave the product with the highest yield. Subsequently, other reaction variables, including the reaction heat and the amount of catalyst were optimized by changing the catalyst amount under various temperatures (Table S1). The results indicated that the highest yield of the model product was achieved at room temperature in the presence of 40 mg catalyst.

The generality of these conditions was then examined Using different raw materials to produce different 1,2,3-Triazoles (Table 2). The results confirm that CuI@HNT-TSC-PC can catalyze the click reaction of all substrates to achieve the corresponding 1,2,3-triazole compounds within short reaction times and in high yields.

**Table 2:**
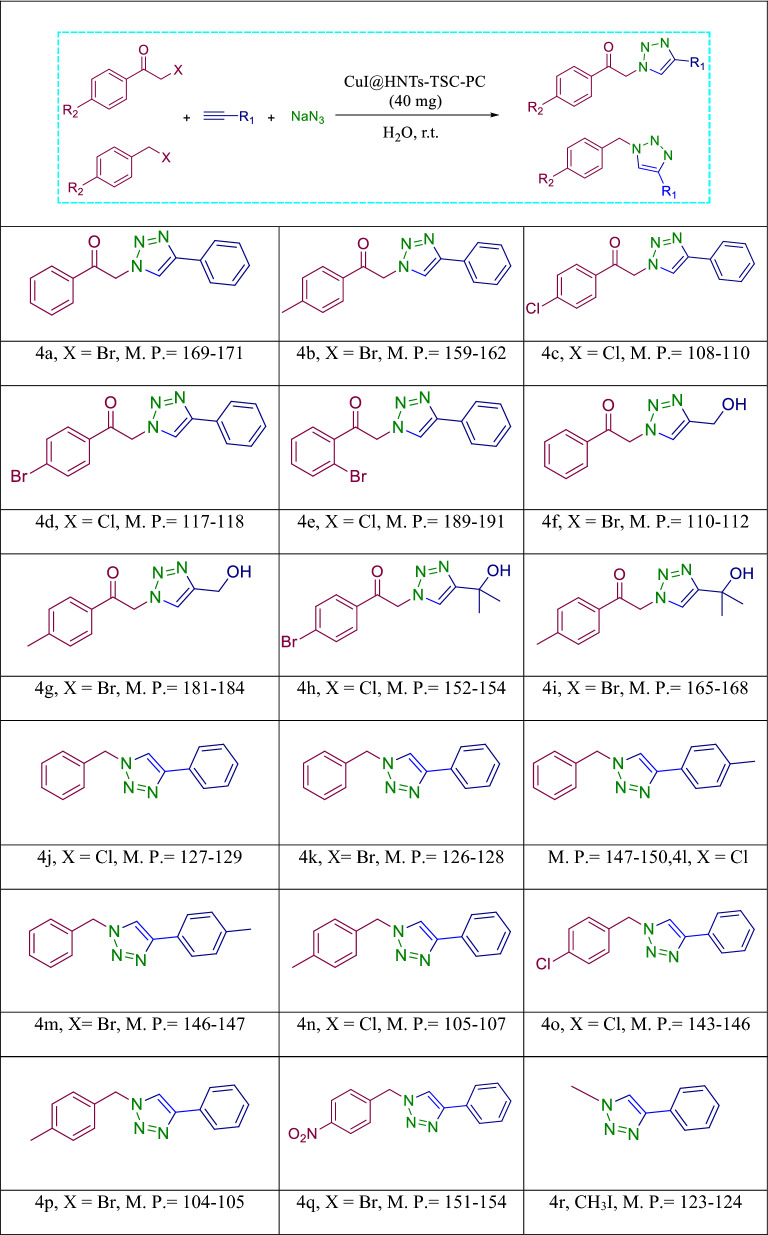
Synthesis of triazole derivatives in the presence of CuI@HNT-TSC-PC^41–43^.

### Catalyst recyclability

To achieve a comprehensive study, the recyclability and copper leaching of this catalyst were surveyed (Fig. S1). The importance of such determinations is because of clarifying the heterogeneous nature of the catalyst and its potential to be used in scale-up processe in industries. Therefore, the yield of the model reaction was determined in the presence of both fresh CuI@HNT-TSC-PC and the recycled ones. It was found that the catalyst can be used up to 6 cycles giving the product with no significant drop of yield. It proves high recyclability of CuI@HNT-TSC-PC.

## Conclusion

In summary, a novel heterogeneous nano composite, CuI@HNT-TSC-PC, was designed and prepared through Cl-functionalization of HNTs followed by reaction with Schiff base and formation of imine functionality and incorporation of CuI. The catalyst was successfully used for promoting Click reaction of α-haloketone or alkyl halide, terminal alkyne and sodium azide in aqueous media and mild reaction condition for the synthesis of 1,2,3-triazoles. Notably, the results of recyclability of the catalyst confirmed its good recyclability and low leaching of Cu species. Moreover, the above-mentioned catalyst was reused up to 6 cycles with slight descent of product yield and Cu leaching.

## Supplementary Information

Supplementary Information.

## Data Availability

The raw/processed data that supports the findings of this study is available from the corresponding author upon reasonable request.
