# Supplementary material for: Investigation of halloysite nanotubes and Schiff base combination with deposited copper iodide nanoparticles as a novel heterogeneous catalytic system
Source: Sci Rep. 2021 Dec 8;11:23658. doi: 10.1038/s41598-021-02991-9 (PMC8654983; doi:10.1038/s41598-021-02991-9)
Supplement: Supplementary file 1 — Supplementary Information. [file 41598_2021_2991_MOESM1_ESM.pdf]

Supporting information

Investigation of halloysite nanotubes & Schiff base combination with deposited copper iodide nanoparticles as a novel heterogeneous catalytic system

*Mansoureh Daraie, Donya Bagheri, Masoume Malmir, Majid M. Heravi**

Department of Chemistry, School of Physics and Chemistry,, Alzahra University, PO Box 1993891176, Vanak, Tehran, Iran.

Tel: (+98) 21-88041347. Fax: (+98) 21-88613935. E-mail: mmheravi@alzahra.ac.ir

Table S1
Optimization of reaction conditions in the Click reaction of phenyl acetylene, benzylbromide and sodium azide[a]

Entry	Loading of Catalyst (mg)	Condition (solvent/ temperature °C)	Time (min)	Yield[b] (%)
1	40	H_2O/ r.t.	16	92
2	40	H_2O/ 50	16	80
3	40	H_2O/ Reflux	16	72
4	50	H_2O/ r.t.	16	92
5	30	H_2O/ r.t.	16	83
6	20	H_2O/ r.t.	16	70
7	None	H_2O/ r.t.	60	25
8	40	H_2O:EtOH (1:1)/ r.t.	16	80
9	40	EtOH / r.t.	16	70
10	40	CH_2Cl_2/ r.t.	16	55
11	40	Toluene / r.t.	16	47
12	40	CH_3CN/ r.t.	16	63
13	40	Solvent free/ r.t.	16	40

[a] Reaction were run in 5 mL solvent with phenyl acetylene (1.0 equiv.), benzylbromide (1.0 equiv.) and sodium azide (1.3 equiv.).
[b] Isolated yield

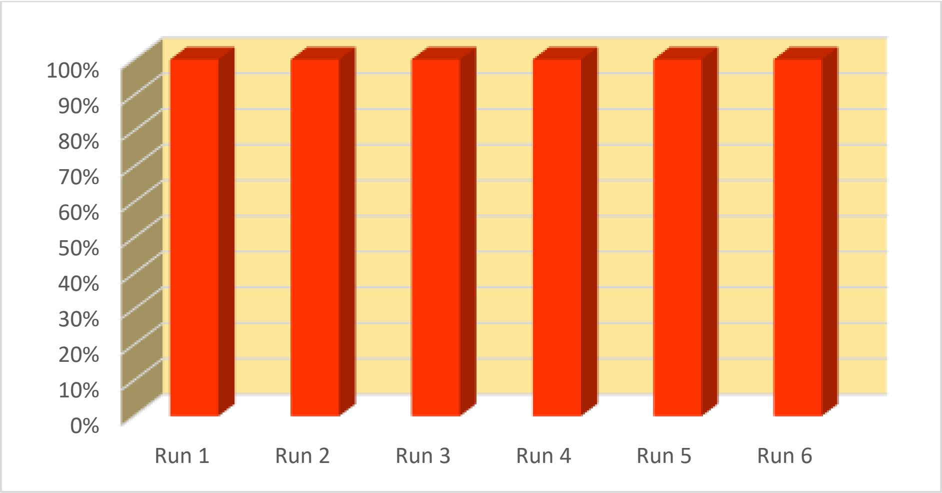

Fig. S1. Reusability of the Cu@HNT-TSC-PC.

1. Spectral data for selected compounds

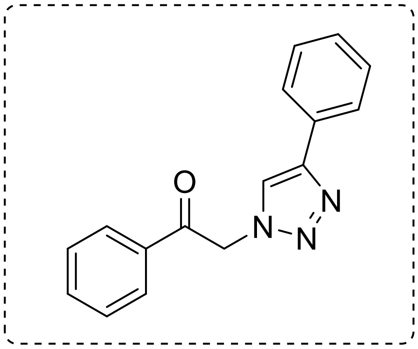

1-phenyl-2-(4-phenyl-1*H*-1,2,3-triazol-1-yl)ethan-1-one (Table 2, 4a): Pale yellow powder; mp. 169-171; υ: 1701, 1223 cm^{-1}; ^{1}H NMR (500 MHz, DMSO) δ: 8.54 (s, 1H, CH-triazole), 8.12-7.34 (m, 10H, Ar), 6.28 (s, 2H, CH_2) ppm; ^{13}C NMR (125 MHz, DMSO) δ: 193.0, 147.2, 135.1, 135.0, 131.6, 129.8, 129.8, 129.1, 128.7, 126.0, 123.9, 56.8 ppm.

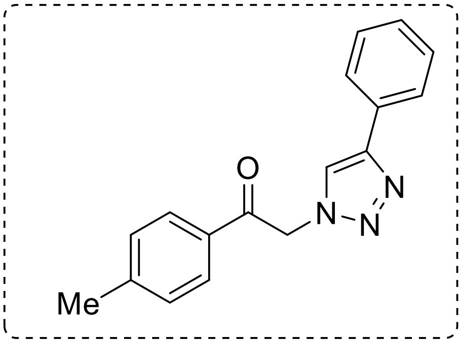

2-(4-phenyl-1*H*-1,2,3-triazol-1-yl)-1-(p-tolyl)ethan-1-one (Table 2, 4b): white powder; mp. 159-162; υ: 1695, 1232 cm^{-1}; ^{1}H NMR (500 MHz, DMSO) δ: 8.52 (s, 1H, CH-triazole), 8.00-7.99 (m, 2H, Ar), 7.88-7.87 (m, 2H, Ar), 7.47-7.33 (m, 5H, Ar), 6.21 (s, 2H, CH_2), 2.41 (s, 3H, CH_3) ppm; ^{13}C NMR (125 MHz, DMSO) δ: 192.4, 147.1, 145.7, 132.5, 131.6, 130.4, 129.8, 129.1, 128.7, 126.0, 123.9, 56.7, 22.1 ppm.

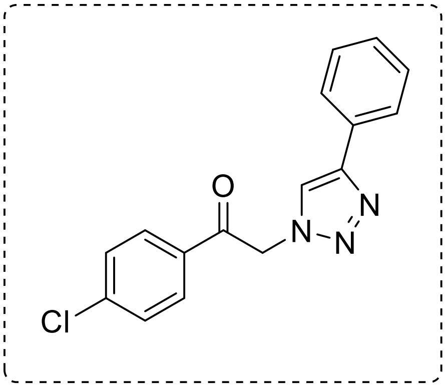

1-(4-chlorophenyl)-2-(4-phenyl-1*H*-1,2,3-triazol-1-yl)ethan-1-one (Table 2, 4c): white powder; mp. 108-110; υ: 1701, 1230 cm^{-1}; ^{1}H NMR (500 MHz, DMSO) δ: 8.52 (s, 1H, CH-triazole), 8.12-7.87 (m, 4H, Ar), 7.71-7.34 (m, 5H, Ar), 6.28 (s, 2H, CH_2) ppm; ^{13}C NMR (125 MHz, DMSO) δ: 192.2, 147.1, 140.0, 133.6, 131.5, 131.0, 130.0, 129.8, 128.7, 126.0, 123.8, 56.8 ppm.

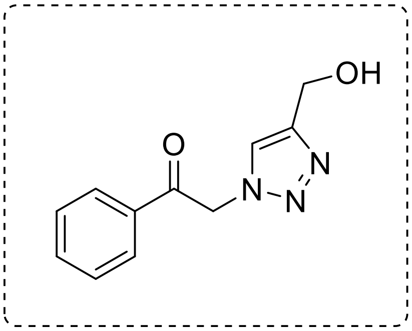

2-(4-(hydroxymethyl)-1*H*-1,2,3-triazol-1-yl)-1-phenylethan-1-one (Table 2, 4f): white powder; mp. 110-112; υ: 3389, 1699, 1229 cm^{-1}; ^{1}H NMR (500 MHz, DMSO) δ: 8.14 (s, 1H, CH-triazole), 8.06-8.05 (m, 2H, Ar), 7.75-7.72 (m, 1H, Ar), 7.62-7.59 (m, 2H, Ar), 6.23 (s, 2H, CH_2), 5.39 (s, 1H, OH), 4.66 (s, 2H, CH_2) ppm; ^{13}C NMR (125 MHz, DMSO) δ: 192.5, 135.1, 134.8, 129.8, 129.0, 57.3, 55.8 ppm.

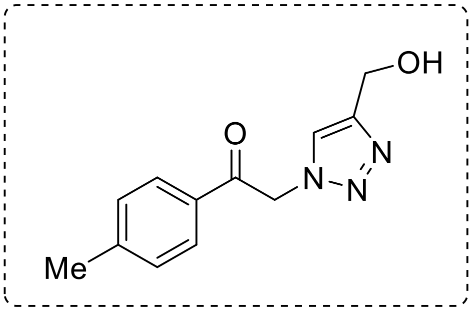

2-(4-(hydroxymethyl)-1*H*-1,2,3-triazol-1-yl)-1-(p-tolyl)ethan-1-one (Table 2, 4g): white powder; mp. 181-184; υ: 3420, 1696, 1235 cm^{-1}; ^{1}H NMR (500 MHz, DMSO) δ: 8.04 (s, 1H, CH-triazole), 7.96-7.95 (m, 2H, Ar), 7.41-7.40 (m, 2H, Ar), 6.13 (s, 2H, CH_2), 5.27 (s, 1H, OH), 4.58 (s, 2H, CH_2), 2.41 (s, 3H, CH_3) ppm; ^{13}C NMR (125 MHz, DMSO) δ: 192.4, 145.6, 132.5, 130.3, 129.1, 56.7, 55.9, 22.1 ppm.

*1.1.*Copies of FTIR, ^{1}H and ^{13}C NMR for selected products

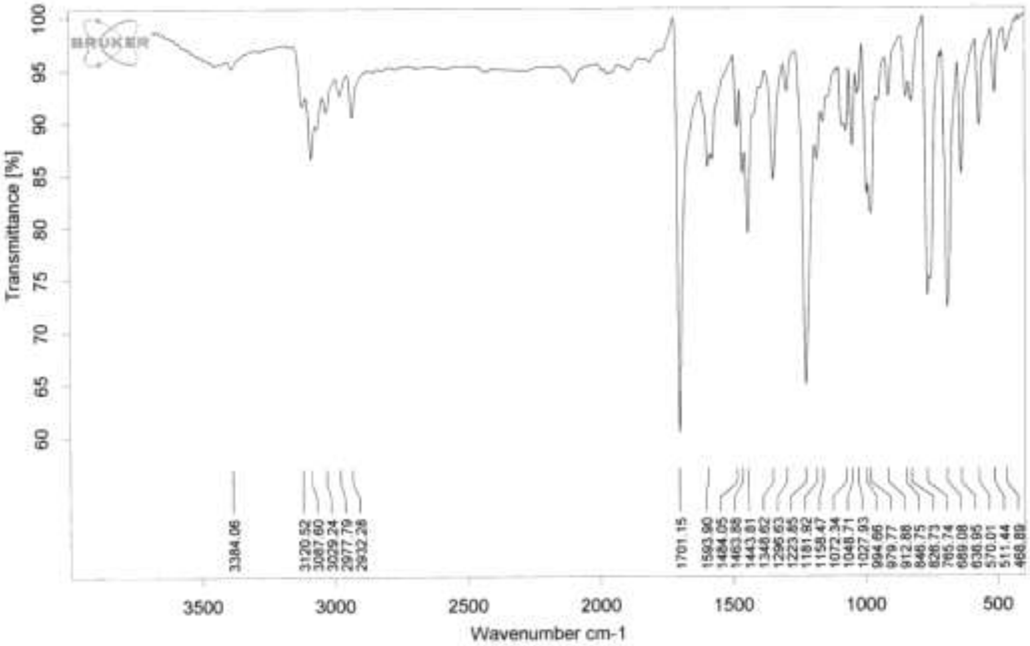

Fig. S2 FTIR spectra of **(Table 2, 4a)**

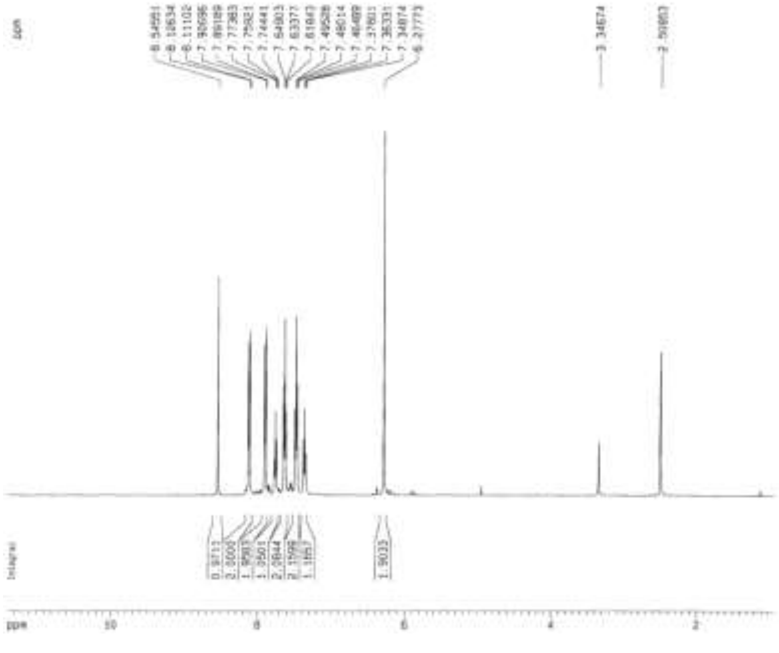

Fig. S3^{1}H NMR spectra of **(Table 2, 4a)**

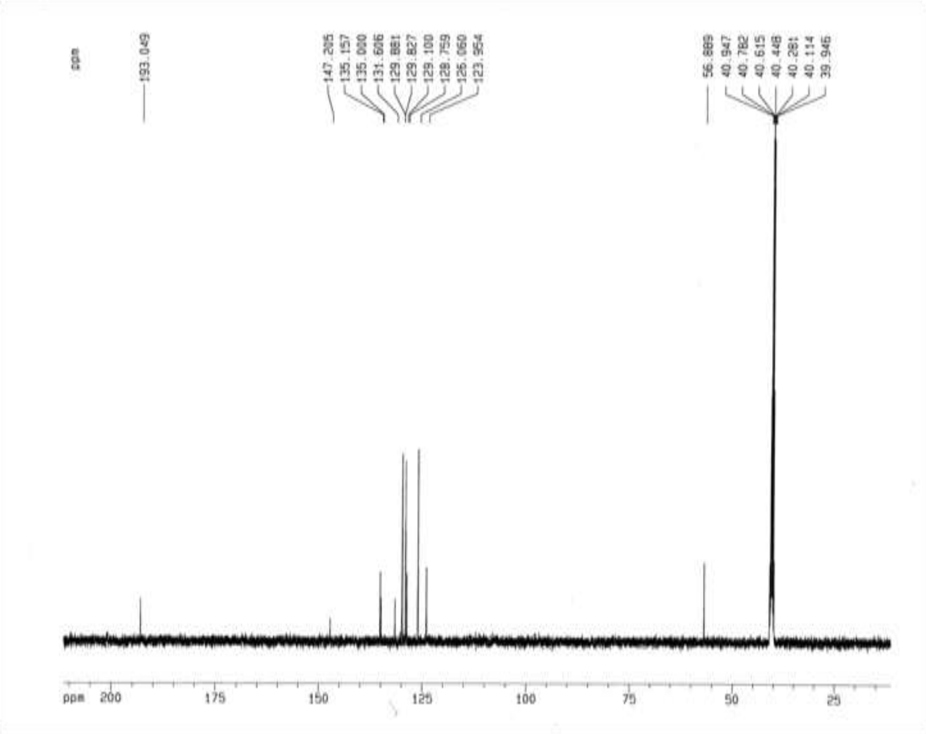

Fig. S4 ^{13}C NMR, spectra of **(Table 2, 4a)**

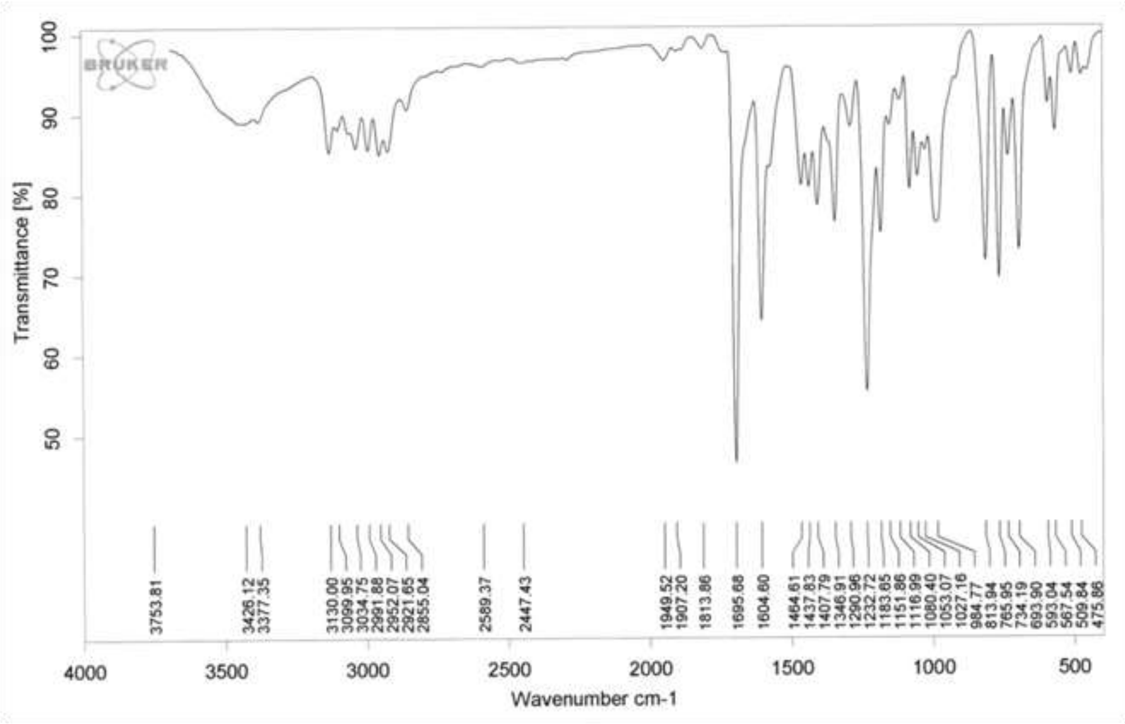

Fig. S5 FTIR spectra of **(Table 2, 4b)**

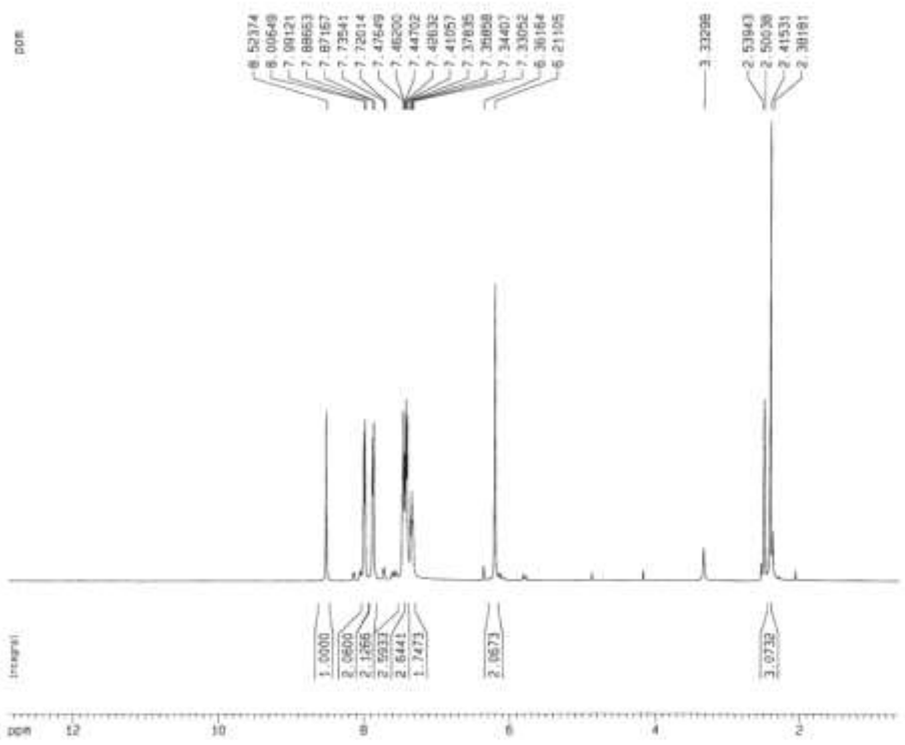

Fig. S6 ^{1}H NMR, spectrum of **(Table 2, 4b)**

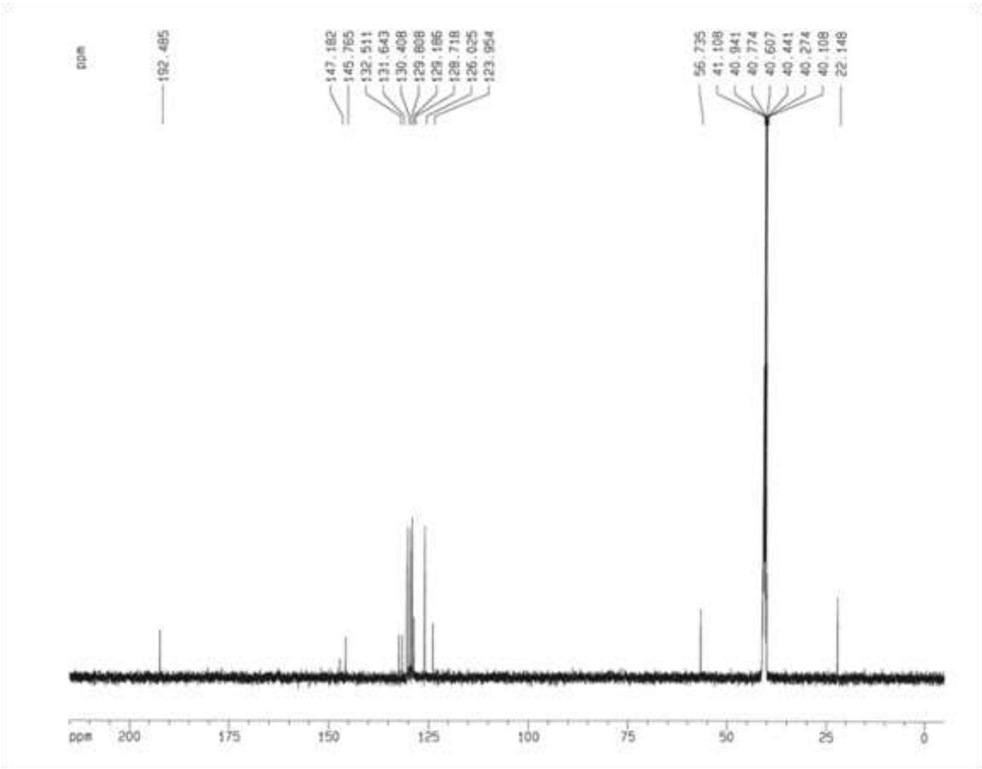

Fig. S7 ^{13}C NMR, spectra of **(Table 2, 4b)**

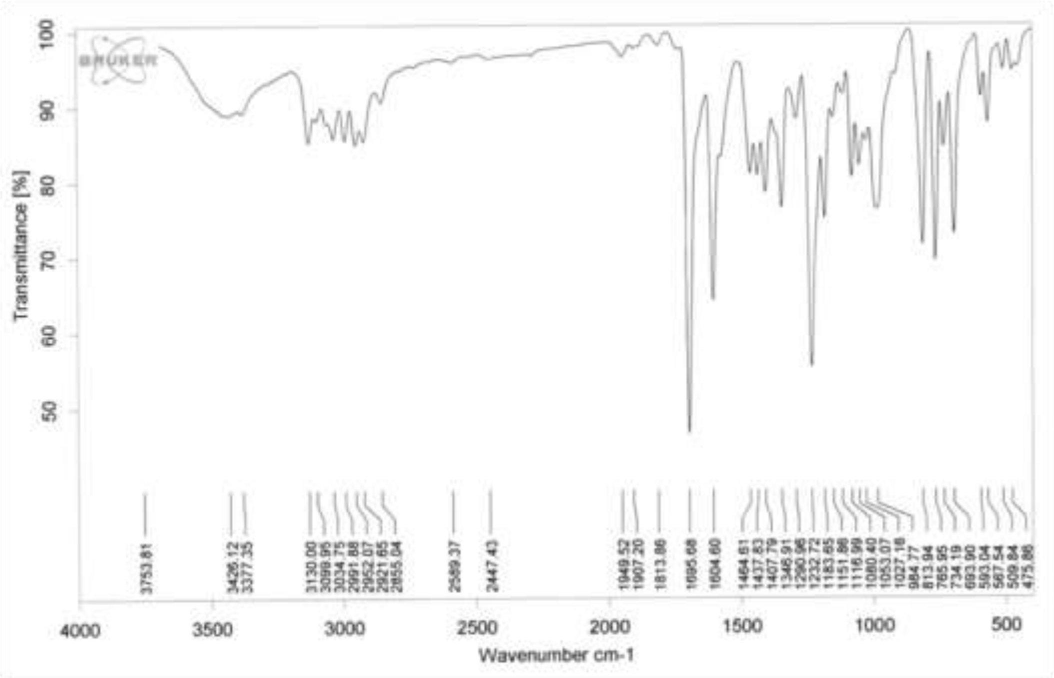

Fig. S8 FTIR spectra of **(Table 2, 4c)**

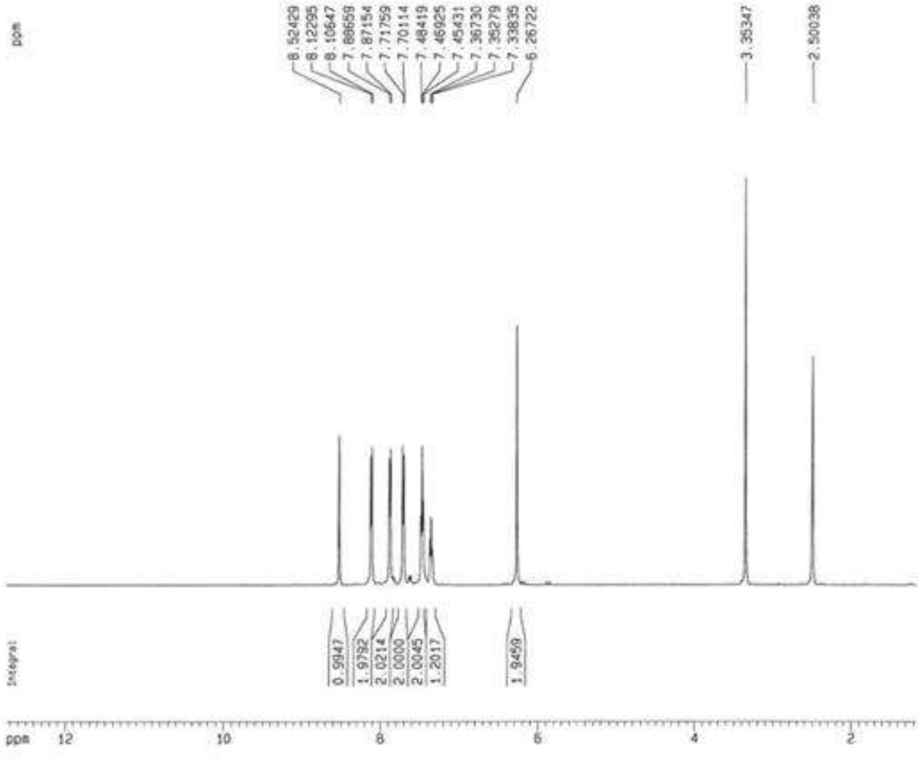

Fig. S9 ^{1}H NMR, spectra of **(Table 2, 4c)**

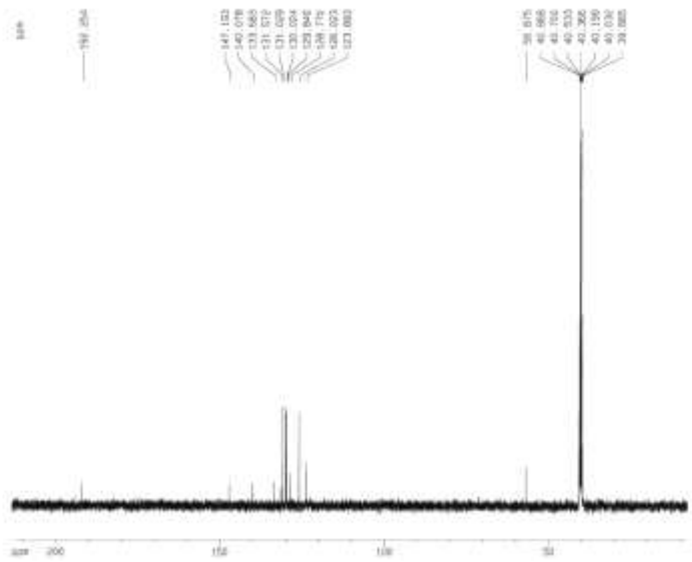

Fig. S10 ^{13}C NMR, spectra of **(Table 2, 4c)**

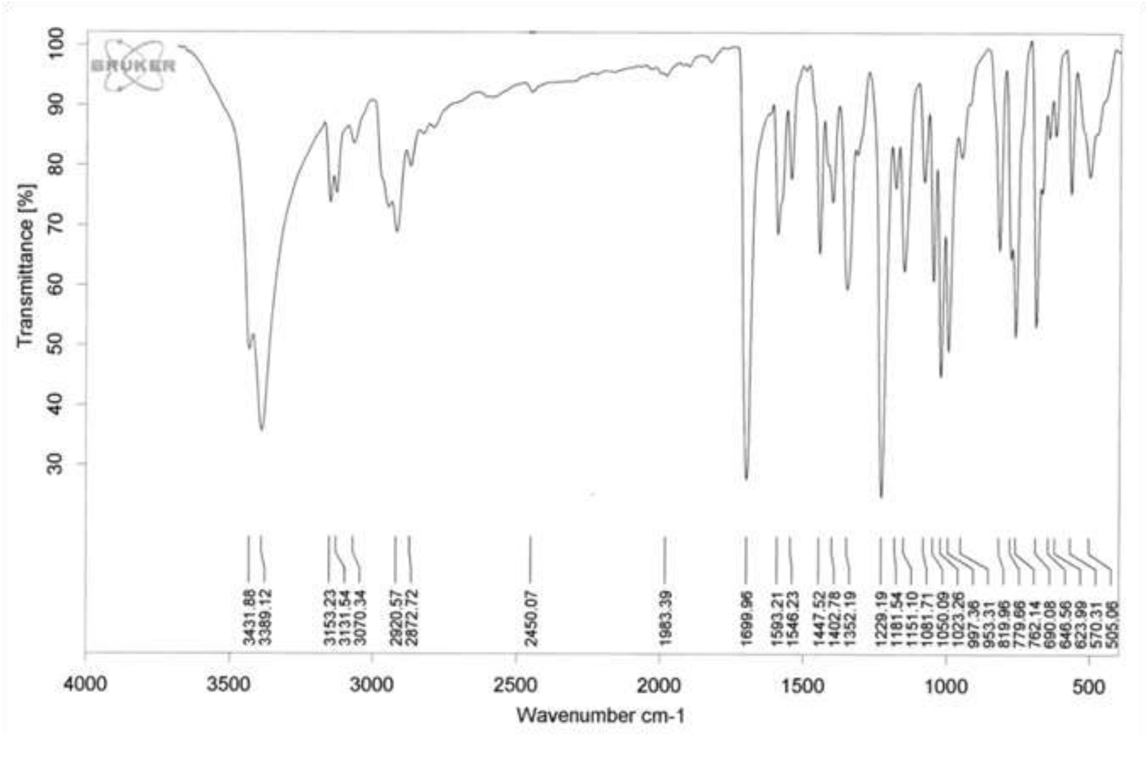

Fig. S11 FTIR spectra of **(Table 2, 4f)**

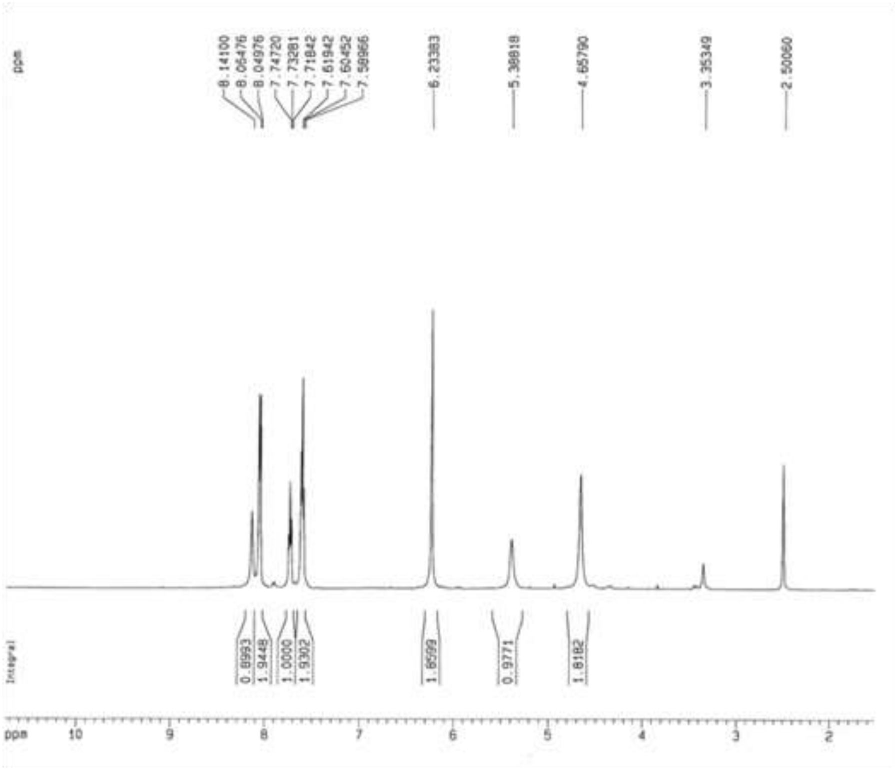

Fig. S12 ^{1}H NMR, spectra of **(Table 2, 4f)**

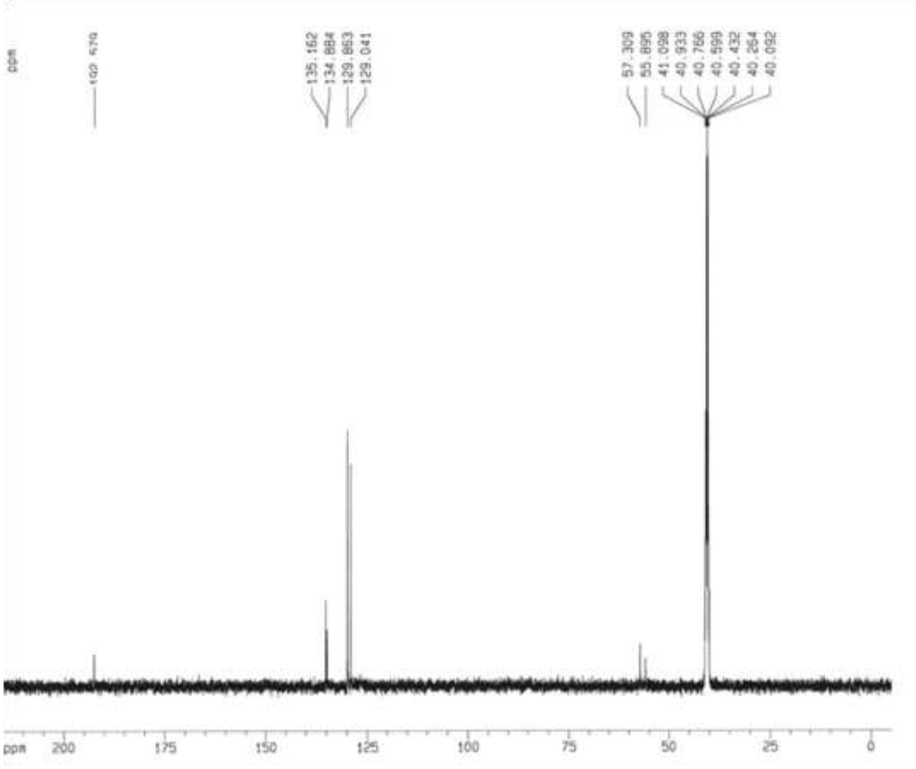

Fig. S13 ^{13}C NMR, spectra of **(Table 2, 4f)**

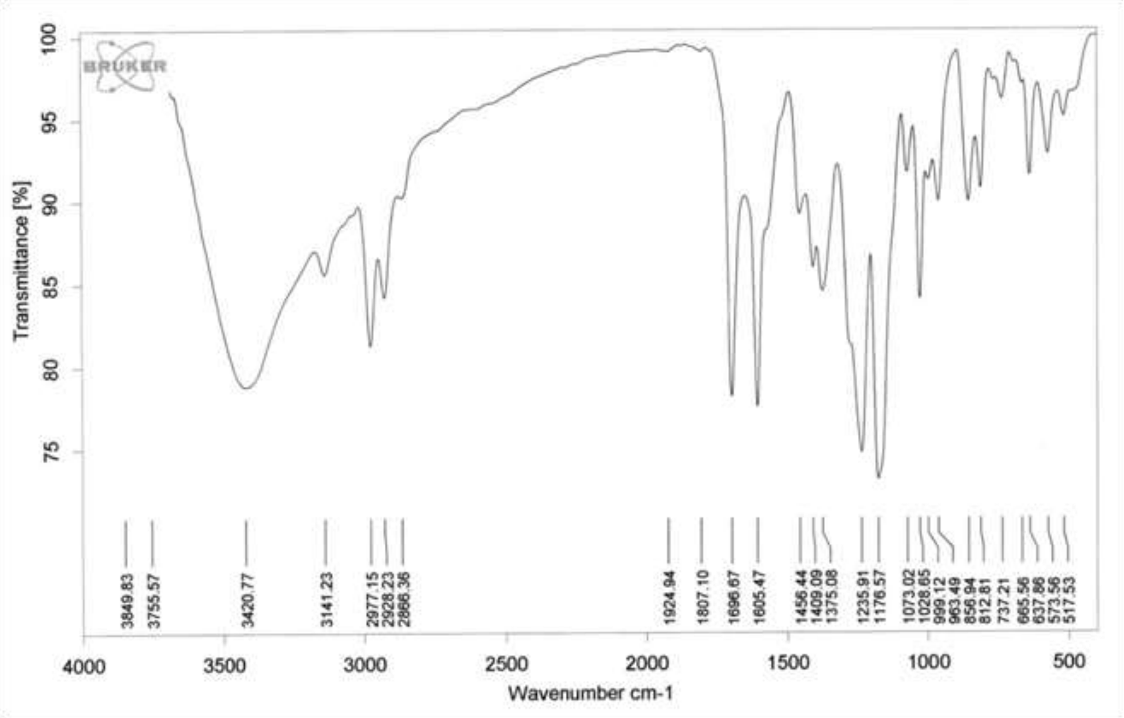

Fig. S14 FTIR spectra of **(Table 2, 4g)**

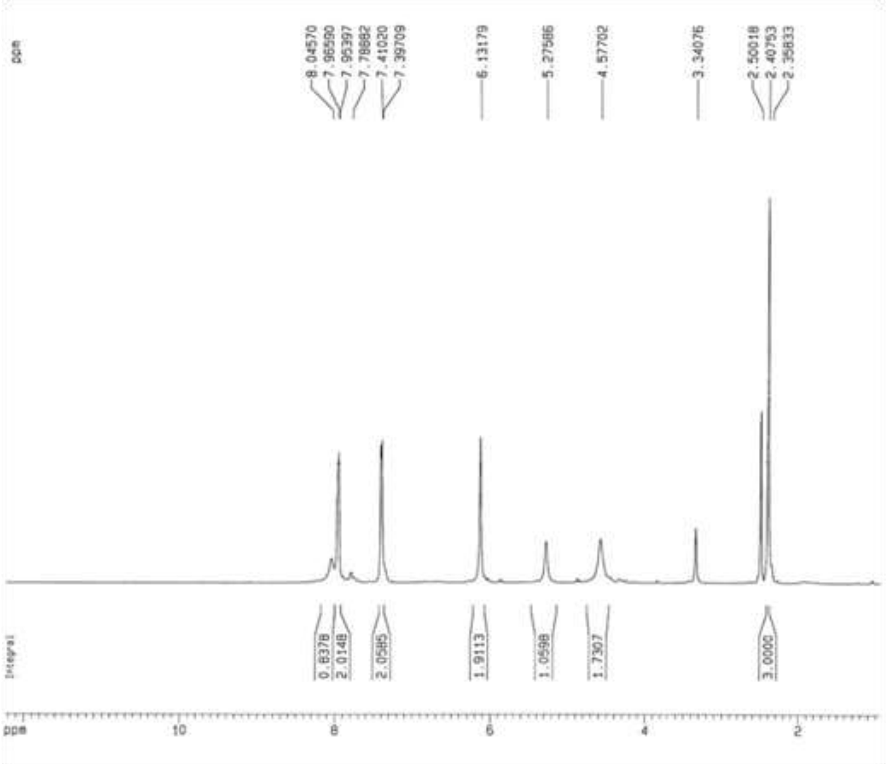

Fig. S15 ^{1}H NMR, spectra of **(Table 2, 4g)**

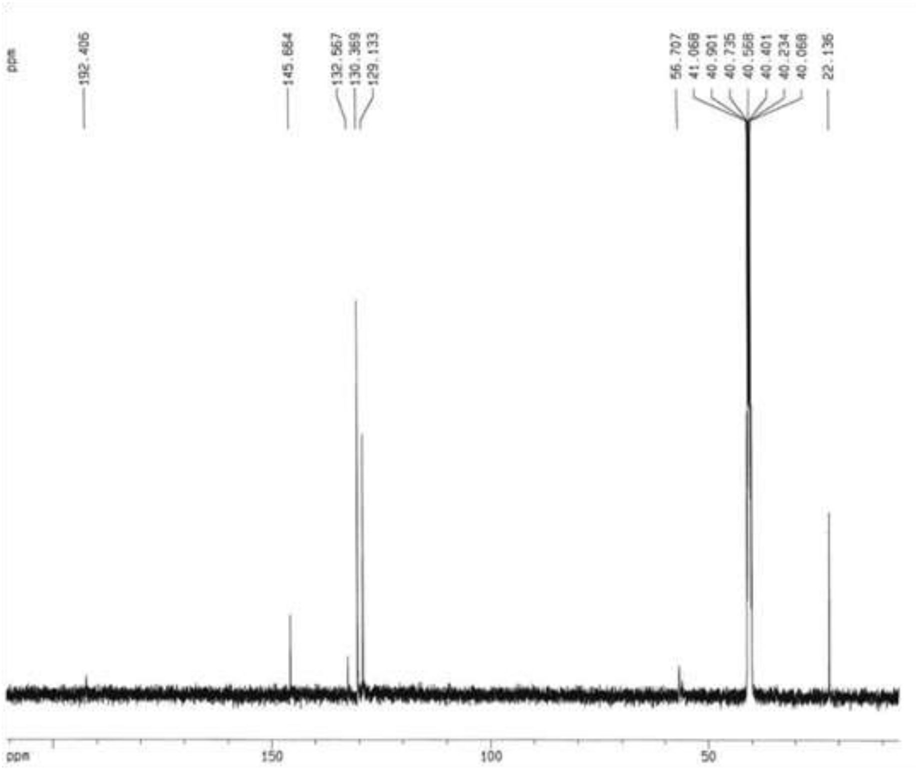

Fig. S16 ^{13}C NMR, spectra of **(Table 2, 4g)**
